# Leukemia risk in children exposed to benzene and PM_10_ from vehicular traffic: a case–control study in an Italian population

**DOI:** 10.1007/s10654-012-9727-1

**Published:** 2012-08-15

**Authors:** Marco Vinceti, Kenneth J. Rothman, Catherine M. Crespi, Antonella Sterni, Andrea Cherubini, Luisa Guerra, Giuseppe Maffeis, Enrica Ferretti, Sara Fabbi, Sergio Teggi, Dario Consonni, Gianfranco De Girolamo, Alessandro Meggiato, Giovanni Palazzi, Paolo Paolucci, Carlotta Malagoli

**Affiliations:** 1 CREAGEN, Environmental, Genetic and Nutritional Epidemiology Research Center, Department of Public Health Sciences, University of Modena and Reggio Emilia, Via Campi 287, 41125 Modena, Italy; 2 Department of Epidemiology, Boston University School of Public Health, Boston, MA USA; 3 RTI Health Solutions, Research Triangle Park, Durham, NC USA; 4 Department of Biostatistics, University of California Los Angeles Fielding School of Public Health, Los Angeles, CA USA; 5 ARPA, Emilia Romagna Region Environmental Protection Agency, Section of Modena, Modena, Italy; 6 TERRARIA srl, Milan, Italy; 7 LARMA, Laboratory of Analysis, Surveying and Environmental Monitoring, University of Modena and Reggio Emilia, Modena, Italy; 8 Unit of Epidemiology, Fondazione IRCCS Ca’ Granda, Ospedale Maggiore Policlinico, Milan, Italy; 9 Department of Public Health, Local Health Unit of Modena, Modena, Italy; 10 Mobility Department, Municipality of Reggio Emilia, Reggio Emilia, Italy; 11 Paediatric Oncohaematology Section, Department of Mother and Child, University of Modena and Reggio Emilia, Modena, Italy

**Keywords:** Childhood leukemia, Benzene, PM_10_, Case–control study, Traffic

## Abstract

Benzene, a recognized occupational leukemogen in adults, has been hypothesized to also increase the risk of childhood leukemia. We carried out a population-based case–control study in a northern Italy community involving 83 cases with acute childhood leukemia diagnosed in the years 1998–2009 and 332 matched controls. We assessed residential exposure to benzene and to particulate matter ≤10 μm (PM_10_) from motorized traffic using geocoded residences and detailed emission and dispersion modeling. Exposure to benzene, and to a lesser extent to PM_10_, appeared to be independently associated with an excess leukemia risk. When we stratified the study population by age and by leukemia subtype, the relative risk associated with benzene exposure was higher among children aged less than 5 years, and despite small numbers this relation appeared to be considerably stronger for acute myeloid leukemia than for acute lymphoblastic leukemia. Overall, these findings suggest that exposure to low levels of benzene released from motorized traffic may increase the risk of childhood leukemia, and suggest a possible independent effect of PM_10_, although unmeasured confounding due to other pollutants cannot be ruled out.

## Background

Benzene has been recognized as a leukemogen, for acute myeloid leukemia in adults and has been associated with other disease subtypes including chronic lymphocytic leukemia and childhood leukemia [1–6]. Increased leukemia risk has been reported at very low environmental levels of benzene [7, 8]. Emissions from motorized traffic, which include benzene, have been hypothesized to increase the risk of childhood leukemia, based on results from recent epidemiologic and toxicological studies [3, 9–13]. This relation, however, is far from accepted, because (1) there are few studies; (2) some studies show null results [14]; (3) there may be uncontrolled confounding; (4) results are generally imprecise; (5) in many locations environmental benzene concentrations have decreased while disease rates have increased; and (6) exposure assessments have had significant limitations. Numerous factors influence ambient air concentrations of pollutants emitted from vehicular traffic. These include diurnal variation in the number and types of vehicles on roads, fuel types, local meteorology, seasonal variation in meteorological conditions, and contributions of emissions from multiple roadways [15–17]. Another important factor is the rapid decrease in concentrations of pollutants emitted by motorized traffic with distance from the roadway, which makes accurate location information important. Few studies have been able to address all these issues, and none seems to have considered the independent effect of benzene taking into account another pollutant of major importance, respirable particulate matter, a key source of exposure to adsorbed toxic and carcinogenic contaminants such as polycyclic aromatic hydrocarbons and heavy metals [18–21].

To address these issues, we conducted a case–control study of childhood leukemia in a northern Italy population that included detailed exposure assessment based on modeling ambient air concentrations of benzene from traffic emissions at the geocoded residence of each subject, together with assessment of exposure to PM_10_ (particles with a diameter of 10 μm or less).

## Methods

### Subjects

This study was a population-based case–control study carried out in the provinces of Modena and Reggio Emilia, both located in the Emilia-Romagna region of northern Italy. These neighboring provinces have a population of about 1,200,000 inhabitants who are homogeneous with respect to ethnicity and socioeconomic status. The cases comprised all children aged 0–14 diagnosed with leukemia while residing in Modena or Reggio Emilia during the years 1998–2009. Cases were identified using the hospital-based registry of the Associazione Italiana Ematologia Oncologia Pediatrica (AIEOP) [22], to which children diagnosed with neoplasms admitted to nearly all Italian hospitals are referred. In the Emilia-Romagna region all four hospitals that treat pediatric neoplasms are included in the AIEOP Registry. Fourteen patients residing in 32 sparsely populated mountain municipalities were excluded from the analysis because of difficulties related to exposure assessment (see following section). The registry database allowed identification of leukemia subtype. Residence at diagnosis was retrieved from administrative databases, mainly consisting of hospital discharge or clinical records.

The control series comprised four subjects matched to each case for sex, year of birth and province of residence during the diagnosis year. Controls were obtained from population data from the National Health Services Local Health Units of Modena and Reggio Emilia, which record all residents annually, by randomly sampling four children among possible matches. For Modena province, no historical population database was available for years before 2005. Therefore, for the selection of Modena province controls for years 1998–2004, we used the 2005 database, and verified actual residence in the year of diagnosis of the matched case through the Revenue Agency of the Ministry of Finance, which maintains records of historical residence nationwide. Additional controls were selected when one or more of the initial selections resided outside the province. Subjects residing in the mountain municipalities located in the southern part of the two provinces were excluded from the population databases before carrying out the sampling procedure. Access to the Ministry of Finance database also allowed us to retrieve annual gross income of parents of the study subjects, which was used to compute family income category for the index year.

### Exposure assessment

We assessed benzene and PM_10_ exposure for each study subject by geocoding his/her residential address and modeling ambient air concentrations at this location. We geocoded residences of cases at date of diagnosis and of controls in the corresponding year using ARC-GIS software (version 9.2, ESRI, Redlands, CA 2006). To geocode addresses we used a database of satellite coordinates made available by Modena and Reggio Emilia provinces or, for addresses not included in the database, Google Earth or a direct *in loco* measure using a portable GPS device (GPSmap 60CSx, Garmin Int. Corp., Olathe, KS). Geocoding also allowed, for subjects residing in the two main study area cities (Modena and Reggio Emilia), evaluation of exposure to magnetic fields at intensity ≥0.1 μT generated from high-voltage power lines, using previously described methodology [23].

The CAlifornia LINE Source Dispersion Model, version 4 (CALINE4), a line source air quality model developed by the California Department of Transportation, was used to model the dispersion of emissions from vehicular traffic. CALINE4 is a stationary plume dispersion model for roads and other linear sources that is used to estimate the dispersion and deposition of pollutants such as carbon monoxide, particulate matter, nitrogen dioxide, benzene and other contaminants at predefined spatial receptors [24]. We entered benzene emissions from vehicular traffic estimated from traffic flows on the main roads of the province. The model was applied over a full year, to encompass daily, weekly, and seasonal variation in weather and traffic conditions. The model predicted hourly benzene concentrations at the location of residence of each study subject at a height of 2 meters. We summarized the final output of the model as an average concentration and a maximum hourly concentration.

We used estimates of traffic flow from previously conducted studies for the province of Modena for the year 2006 and for the province of Reggio Emilia for 2005 [25, 26]. The traffic flow estimates were generated using a model that incorporated demographic and occupational information for all residents of the provinces, and detailed personal mobility information collected by the National Institute of Statistics 2001 Census and validated through surveys and with automatic vehicles counters. The model created a matrix of vehicle movements for each road, on the basis of daily movements estimated for their residents taking into account their age, sex, family structure and occupation [25, 26]. For Reggio Emilia, these data were further validated by a survey of randomly selected families and car drivers carried out in 2005 by the Department of Planning of Venice University, coordinated by one of the authors (A.M.) [26].

We computed emissions using emission factors for light and heavy vehicles and for urban and suburban areas. The emission factors for benzene were derived from a 1990–2007 transport database developed by the Italian National Institute for Environmental Protection and Research (www.isprambiente.gov.it) and calculated using the program COPERT IV developed by the Laboratory of Applied Thermodynamics of the Aristotle University of Thessaloniki (www.emisia.com/copert/General.html). The COPERT IV emission factors are detailed by directive reference, engine capacity, weight class and fuel, for different vehicles classes (passenger cars, light duty vehicles, heavy duty vehicles, urban buses and coaches, two wheelers) and tabulated according to the driving cycle (urban, suburban and highway, given the dependence of the emission factors of vehicle speed). Mean values of benzene emission factors (calculated from the number of vehicles registered and from the relative annual average mileage) were 23.5 and 0.82 mg/km for light and heavy vehicles, respectively, in the urban cycle, and 2.96 and 0.31 mg/km for rural cycle.

Meteorological data were obtained using a meteorological model, CALMET, deployed at Hydro Meteorological Service of the Emilia-Romagna environmental protection agency ARPA—Agenzia Regionale per la Protezione Ambientale (www.arpa.emr.it/cms3/documenti/_cerca_doc/meteo/ambiente/descrizione_calmet.pdf). This model elaborates, on the basis of measured data, parameters such as temperature, wind speed and direction, stability class and height of the mixing layer.

Using the above data, we used CALINE4 to estimate average benzene concentration for each hour of the simulation year at each study subject’s location. As suggested by the CALINE4 Technical Guide [24], we ran the model increasing the road width by 3 meters to the right and left, in order to account for thermal and mechanical turbulence caused by vehicles. We removed from further analysis the mountain areas located in the southern part of the two provinces, due to rough topography, which cannot be taken in account by CALINE4. Less than 10 % of the total population of the two provinces resided in the excluded area.

Some simplifications were incorporated into the modeling. We did not consider the effect of additional turbulence created by tall buildings (urban canyons) because we lacked information about building height. Since the calculation domain was located mainly in the flat area of the two provinces, the land was considered to be flat. No additional emission sources such as industrial solvent use, parking lots or petrol stations were considered.

We modeled ambient air concentrations of PM_10_ released from traffic using the same methodology as described for benzene. PM_10_ emission factors include both exhaust (emissions from tailpipe, obtained from ISPRA database based on COPERT IV calculations) and non-exhaust components (abrasion and resuspension processes, obtained from Gehrig determination [27]). Values of PM_10_ emissions factors used in our calculations were 105.7 and 1,054.6 mg/km for light and heavy vehicles, respectively, in the urban cycle, and 62.3 and 337.0 mg/km for rural cycle.

To validate the results, we ran the CALINE4 model with reference to 3 air quality monitoring stations in Reggio Emilia in 2005 and 4 monitoring stations in Modena in 2006; one station was excluded due to unreliability of traffic data for that location. The Pearson correlation coefficient between the estimated (modeled) and measured yearly mean levels (maximum measured levels were not available for technical reasons) was 0.43 (95 % CI −0.48–0.89) for benzene and for 0.64 (95 % CI −0.21–0.94) for PM_10_. Historical data from these monitoring stations were also used to characterize trends in ambient air levels of benzene over the study period; results indicated a decrease in benzene concentrations over time, with average concentrations in the 1998–2003 period about twice the values for 2004–2009; for the only Modena city monitoring station operating throughout the entire study period, for example, values averaged 5.2 μg/m^3^ for 1998–2003 and 2.2 μg/m^3^ for 2004–2009. This decrease is consistent with reduction in the benzene content of gasoline over this time period as well as a change in the composition of the vehicle fleet towards lower emission vehicles.

### Statistical analysis

Analyses were conducted using bivariate and multivariate conditional logistic regression models and generalized additive models [28]. To examine the sensitivity of the findings to model specification, we entered exposure in the models using both categorical cutpoints and continuous variables, with the latter modeled as both untransformed and log-transformed. Each generalized additive model consisted of a logistic regression model in which the relation between the log odds of being a case and the exposure variable was modeled nonparametrically using a natural cubic spline, with control for the matching variables (sex, age, year of diagnosis and province) and the other pollutant. Since the distributions of the exposure variables (mean benzene and mean PM_10_ concentrations) were right-skewed with outliers at high exposure levels, log transformations were used to achieve approximately symmetric distributions and reduce the influence of outliers. To improve interpretability, we used a log base 10 transformation, so that a one-unit increase in the log-transformed variable is equivalent to a tenfold increase in exposure (e.g., 0.05 vs. 0.5 μg/m^3^). These analyses were conducted using the gam package in R version 2.9.2 [29]. Other analyses were conducted using Stata 12.1 (Stata Corp. College Station, TX, 2012). We conducted analysis stratified by age of diagnosis (<5 vs. ≥5 years) to explore possible age-related susceptibilities and to minimize the effect of exposure misclassification due to antecedent changes of residence, and subgroups analyses for acute lymphoblastic leukemia and acute myeloid leukemia.

## Results

During the study period, 83 cases of acute childhood leukemia (50 males and 33 females) occurred in the study area, including 64 cases of lymphoblastic leukemia (average age at diagnosis 5.1, standard deviation 4.8 years) and 19 of other leukemia types included in the myeloid category (6.6 ± 3.8 years). The distribution of estimated annual average and maximum hourly benzene and PM_10_ concentrations in outdoor ambient air are reported in Table 1. For the majority of subjects, the estimated average benzene and PM_10_ exposure from vehicular traffic were considerably lower than the European Union (EU) standards for ambient air of 5 and 40 μg/m^3^, respectively. Mean concentrations of benzene and PM_10_ were moderately associated (Pearson correlation coefficient 0.53).

**Table 1:** Distribution of cases and controls by approximate quartiles of annual average and maximum benzene and PM_10_ concentrations in outdoor ambient air (μg/m^3^) among controls

	I	II	III	IV
*Average benzene levels*				
Cutpoints	<0.10	0.10–<0.25	0.25–<0.50	≥0.50
Cases/controls	16/80	18/86	17/75	32/91
*Maximum benzene levels*				
Cutpoints	<2	2–<4	4–<6	≥6
Cases/controls	17/83	21/88	17/80	28/81
*Average PM*_10_ *levels*				
Cutpoints	<2.5	2.5–<5	5–<7.5	≥7.5
Cases/controls	18/91	16/79	21/70	28/92
*Maximum PM*_10_ *levels*				
Cutpoints	<25	25–<50	50–<75	≥75
Cases/controls	6/21	17/104	35/112	25/95

In analyses with exposure categories based on cutpoints yielding nearly equal sized number of controls in four categories, relative risk (RR) values, as estimated through the odds ratios, were elevated in the highest category of benzene exposure, for both average and maximum hourly concentration, both in the crude analysis and when adjusted for average PM_10_ exposure (Table 2). Further adjustment for parental income and, in the urban area of Modena and Reggio Emilia, for exposure to magnetic fields from high-voltage power lines, or assigning exposure status according to exact quartile cutpoints of pollutants exposure among controls, had little effect on the relative risk estimates (data not shown). RR values were also elevated in the two highest categories of average PM_10_ levels, and remained elevated after adjustment for average benzene concentration (Table 2). There was no indication of increased risk associated with maximum hourly PM_10_ levels.

**Table 2:** Odds ratios (OR) with 95 % confidence intervals (CI) of childhood leukemia associated with categories (μg/m^3^) of benzene and PM_10_ exposure (95 % confidence interval) from conditional logistic regression analysis of matched case–control sets

	I	II	III	IV
	Referent	OR (95 % CI)	OR (95 % CI)	OR (95 % CI)
*Average benzene levels*	<0.10	≥0.10–0.25	≥0.25–0.50	≥0.50
Crude	1.0	1.1 (0.5–2.3)	1.2 (0.5–2.7)	1.8 (0.9–3.7)
Adjusted for average PM_10_ exposure	1.0	0.8 (0.5–2.6)	1.1 (0.5–2.6)	1.7 (0.8–3.6)
*Maximum hourly benzene levels*	<2	≥2–4	≥4–6	≥6
Crude	1.0	1.2 (0.6–2.6)	1.1 (0.5–2.2)	1.8 (0.9–3.6)
Adjusted for maximum hourly PM_10_ exposure	1.0	1.1 (0.5–2.5)	0.9 (0.4–2.1)	1.6 (0.7–3.4)
*Average PM*_*10*_ *levels*	<2.5	≥2.5–5	≥5–7.5	≥7.5
Crude	1.0	1.1 (0.5–2.3)	1.6 (0.8–3.4)	1.8 (0.8–3.9)
Adjusted for average benzene exposure	1.0	1.1 (0.5–2.3)	1.6 (0.7–3.5)	1.8 (0.8–3.9)
*Maximum hourly PM*_*10*_ *levels*	<25	≥25–50	≥50–75	≥75
Crude	1.0	0.6 (0.2–1.7)	1.1 (0.4–2.9)	0.9 (0.3–2.7)
Adjusted for maximum hourly benzene exposure	1.0	0.6 (0.2–1.6)	1.0 (0.4–2.9)	0.9 (0.3–2.7)

Results of analyses stratified by age of diagnosis of case (<5 years and ≥5 years) using approximate quartiles of exposure are presented in Table 3. In the younger group, there was an increased RR associated with the third and particularly the fourth quartile of mean benzene exposure, adjusting for PM_10_. RR estimates for PM_10_ adjusted for benzene exposure were also consistent with a dose–response relation. For children aged 5 years or older, RRs associated with average benzene exposure were near unity in all exposure categories, while RRs were higher for average PM_10_ in the third and fourth quartiles of exposure.

**Table 3:** Odds ratios (OR) with 95 % confidence intervals (CI) of childhood leukemia associated with categories of benzene and PM_10_ exposure stratified by age of diagnosis of case from conditional logistic regression analysis of matched case–control sets

	Category			
	I	II	III	IV
	(referent)	OR (95 % CI)	OR (95 % CI)	OR (95 % CI)
Age < 5 years				
Average benzene levels	1.0	1.0 (0.3–3.6)	1.3 (0.4–5.1)	3.3 (1.0–10.3)
Average PM_10_ levels	1.0	1.0 (0.3–3.4)	1.6 (0.5–5.1)	1.9 (0.6–6.0)
Age ≥ 5 years				
Average benzene levels	1.0	1.0 (0.4–2.8)	0.9 (0.3–2.5)	0.9 (0.3–2.5)
Average PM_10_ levels	1.0	1.0 (0.4–2.8)	1.3 (0.4–3.8)	1.5 (0.5–4.9)

Adjusting simultaneously for the other pollutant

**Fig. 1:**
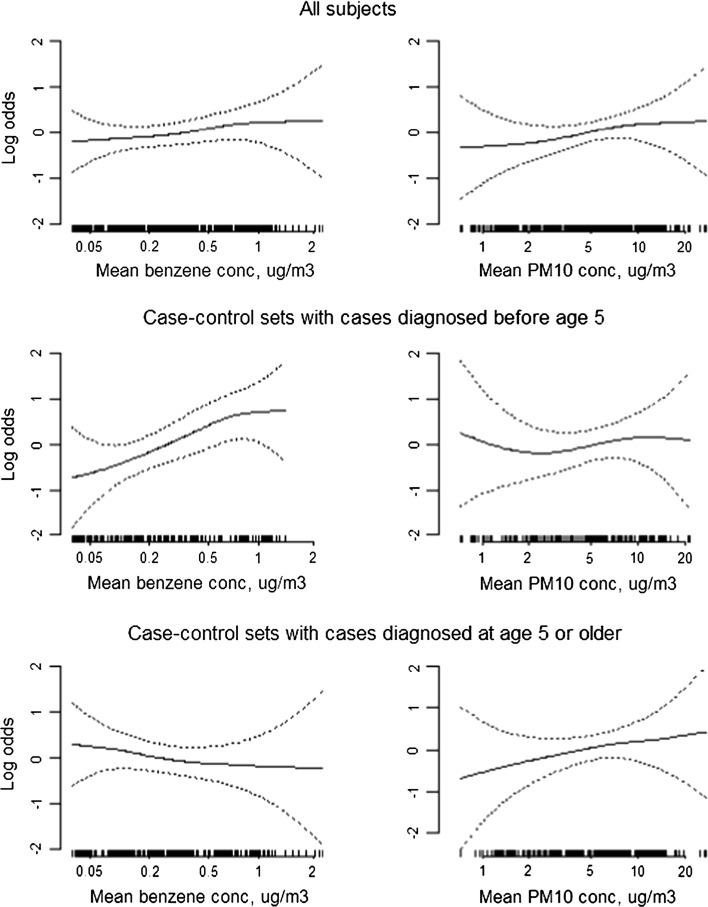
Natural cubic spline models from generalized additive model for the relation between log odds of case status and mean benzene and between case status and PM_10_ exposure, controlling for sex, age, year of diagnosis, province and the other pollutant. *Rugplots* at the *bottom* of each plot provide the distribution of exposure levels of subjects

Nonparametric natural cubic spline regression analysis (Fig. 1), with adjustment for the matching variables, suggested a positive association between average benzene exposure and leukemia risk in the overall population, with adjustment for PM_10_. In age-stratified analyses, there was a strong positive association among children younger than 5 years, and suggestion of a negative association among children 5 years or older. Results for PM_10_ also suggested a possible positive association with leukemia risk in the population overall. In age-stratified analyses, there was little evidence of an association among children younger than 5 years but a possible positive association among children 5 years and older (Fig. 1).

Since the nonparametric analyses suggested that the log odds might be linear in the log-transform of each exposure variable, RRs were estimated for a conventional parametric conditional logistic regression model using log-transformed exposure variables (Table 4), in addition to untransformed values to assess sensitivity of the findings to model specification. In the overall population, a one-unit log base 10-transformed increase in benzene exposure, corresponding to a tenfold increase in mean benzene concentration, was associated with a RR of 1.37 (95 % CI 0.83–2.25), while the corresponding RR for PM_10_ was 1.51 (95 % CI 0.71–3.20). Adjustment for income increased the RRs slightly, while adjustment for the other pollutant attenuated the RR estimates somewhat. Estimates computed using the exposure variables on their original untransformed scale yielded roughly comparable results.

**Table 4:** Odds ratios (95 % confidence intervals) of childhood leukemia associated with an increase in average benzene and PM_10_ concentrations from conditional logistic regression analyses of matched case–control sets

	Untransformed values	Log-transformed values
All subjects		
*Benzene*		
Crude	1.23 (0.72–2.12)	1.37 (0.83–2.25)
Adjusted for income	1.27 (0.73–2.20)	1.38 (0.84–2.31)
Adjusted for PM_10_	1.16 (0.66–2.04)	1.26 (0.71–2.25)
*PM*_10_		
Crude	1.39 (0.79–2.44)	1.51 (0.71–3.20)
Adjusted for income	1.49 (0.84–2.65)	1.62 (0.76–3.46)
Adjusted for benzene	1.34 (0.75–2.40)	1.26 (0.52–3.02)
Subjects <5 years old		
*Benzene*		
Crude	3.07 (1.22–7.75)	2.72 (1.23–6.01)
Adjusted for income	2.72 (1.08–6.86)	2.73 (1.24–5.99)
Adjusted for PM_10_	2.89 (1.12–7.43)	2.77 (1.06–7.27)
*PM*_10_		
Crude	1.62 (0.69–3.82)	2.18 (0.76–6.32)
Adjusted for income	1.58 (0.66–3.78)	2.24 (0.78–6.42)
Adjusted for benzene	1.33 (0.53–3.34)	0.96 (0.25–3.70)
Subjects ≥5 years old		
*Benzene*		
Crude	0.70 (0.31–1.60)	0.86 (0.49–1.52)
Adjusted for income	0.69 (0.31–1.58)	0.86 (0.48–1.52)
Adjusted for PM_10_	0.65 (0.28–1.55)	0.84 (0.46–1.54)
*PM*_10_		
Crude	1.23 (0.58–2.65)	1.00 (0.33–2.98)
Adjusted for income	1.22 (0.56–2.69)	0.97 (0.32–2.96)
Adjusted for benzene	1.36 (0.62–3.01)	1.12 (0.35–3.64)

OR associated with a 1 μg/m^3^ increase in average benzene concentration and with a 10 μg/m^3^ increase in average PM_10_ concentration

When stratified by age, a much higher RR of disease associated with benzene exposure was found for children younger than 5 years, with a RR of 2.72 (95 % CI 1.23–6.01), compared with older children, who had a RR of 0.86 (95 % CI 0.49–1.52). Higher RRs among younger subjects also emerged for PM_10_ but were attenuated after adjustment for benzene exposure. Analyses carried out using the crude benzene and PM_10_ values yielded substantially similar results.

Subgroup analysis according to leukemia type showed much higher estimates, though statistically very unstable, for myeloid leukemia than for lymphoblastic leukemia in the youngest age group (Table 5).

**Table 5:** Odds ratios (95 % confidence intervals) of childhood leukemia associated with average benzene and PM_10_ concentrations according to leukemia subtype, acute lymphoblastic leukemia (ALL) and acute myeloid leukemia (AML)

	ALL (cases = 64)	AML (cases = 19)
All subjects		
*Benzene*		
Crude	1.08 (0.57–2.04)	1.90 (0.63–5.73)
Adjusted for PM_10_	0.97 (0.49–1.93)	1.92 (0.64–5.78)
*PM*_10_		
Crude	1.44 (0.75–2.74)	1.26 (0.40–3.94)
Adjusted for benzene	1.45 (0.73–2.86)	1.30 (0.41–4.16)

	ALL (cases = 27)	AML (cases = 11)
Subjects <5 years old		
*Benzene*		
Crude	2.23 (0.71–7.03)	5.51 (1.13–26.91)
Adjusted for PM_10_	1.95 (0.58–6.50)	5.46 (1.12–26.51)
*PM*_10_		
Crude	1.77 (0.66–4.78)	1.25 (0.23–6.85)
Adjusted for benzene	1.50 (0.52–4.34)	1.21 (0.18–8.19)

	ALL (cases = 37)	AML (cases = 8)
Subjects ≥5 years old		
*Benzene*		
Crude	0.77 (0.32–1.82)	0.42 (0.04–4.83)
Adjusted for PM_10_	0.69 (0.27–1.79)	0.43 (0.04–4.97)
*PM*_10_		
Crude	1.23 (0.51–2.95)	1.26 (0.27–5.88)
Adjusted for benzene	1.39 (0.54–3.57)	1.18 (0.25–5.55)

OR associated with a 1 µg/m^3^ increase in average benzene concentration and with a 10 µg/m^3^ increase in average PM_10 _concentration

## Discussion

Our results suggest the benzene exposure from vehicular traffic at levels lower than the current European Union limit of 5 μg/m^3^ [30] may have been associated with increased risk of childhood leukemia in this Italian population, particularly for children under 5 years of age. Confounding due to socioeconomic factors, magnetic fields exposure (as suggested by Langholtz et al. [14]) and PM_10_ from vehicular traffic did not appear to explain these findings, though the only variable available to assess parental socioeconomic status might not have controlled it adequately. PM_10_ was also associated with elevated RRs in children under the age of 5, but this association was attenuated when adjustment was made for benzene exposure. These results for benzene are consistent with some previously reported epidemiologic studies that examined the relation between exposure from motorized traffic and childhood leukemia risk [12, 31] and with evidence concerning adult lymphoid leukemia and other lymphoid malignancies [3, 7]. Maximum pollutant exposure did not yield additional evidence of a relation with the excess leukemia risk compared with average levels, and this was particularly true for PM_10_, suggesting that usual exposure, the one generally monitored by regulatory agencies, is not less informative than peak exposures in influencing disease risk.

This study has important limitations. The study design did not allow for collection of information directly from subjects and their families, so there is limited information about potential confounders. On the other hand, available evidence from the literature has not consistently identified major risk factors for childhood leukemia, apart from ionizing and non-ionizing radiation and possibly genetic susceptibility [31–33], and there is no reason to hypothesize a different prevalence of these variables across benzene exposure subgroups in our study. Moreover, contacting study subjects would have created a risk of selection and recall bias, and the inclusion of family income and matching on age, sex, year of diagnosis and province should have reduced the risk of confounding due to unmeasured factors. We used modeled ambient air levels to estimate study subjects’ exposure. There were multiple possible sources of error in the exposure assessment, including the emission factors utilized, the vehicle traffic estimates and the use of a single calendar year to represent exposures that occurred at various times during the study period. Owing to the limited number of air monitoring stations available, we had limited ability to validate the modeling, and our validation suggested only moderate correlation between modeled and measured ambient air levels. Errors in measurement of exposure could have introduced bias and imprecision into the effect estimates. However, the measurement error was likely to have been non-differential with respect to disease status and thus could be expected to bias risk estimates towards the null. We attempted to isolate the effect of single agents (benzene and PM_10_) whereas vehicular exhaust is a complex mixture of agents, and unmeasured confounding due to other pollutants may have occurred. We used the residential location at time of diagnosis (or in the corresponding year for referents) to assess benzene exposure for study subjects, an approach that might not have adequately accounted for antecedent exposure due to changes of residence, thus inducing some degree of exposure misclassification. However, in a previous study in which we evaluated long-term historical residence of case and control children residing in Modena and Reggio Emilia municipalities [23], residential mobility was low; 70.3 % (225/320) of children examined in that investigation had never changed residence before the year of each case’s diagnosis; for children aged less 5 years, 82.2 % (152/185) had never changed residence (Malagoli et al., unpublished data). A further limitation was the exclusion of cases residing in mountainous areas owing to the inability of the CALINE4 model to handle rough topography.

When we limited the analysis to subjects younger than 5 years, the RRs associated with benzene exposure were higher than those calculated for the overall study population. The higher RR among these younger subjects could be explained in part by lower exposure misclassification, considering their lower probability of having changed addresses in the past, and by their likelihood of spending more time at home than older children; the percentage of children attending school in the 2003–2004 school year was 27 % and 25 % in the age group 0–3 years in Modena and Reggio Emilia provinces, respectively, rapidly increasing to 97 and 92 %, respectively, for age 5 (courtesy of Emanuela Bertozzi and Margherita Malagoli from the Reggio Emilia and the Modena Province Authorities). This higher RR is also compatible with the hypothesis that younger children have a greater susceptibility to adverse effects of benzene released by motor vehicles, not an entirely unexpected finding since some chemicals may pose a higher risk of cancer when exposure occurs during early life [34]. Several previous epidemiologic studies carried out age-specific subgroup analyses [9, 35–38]. Savitz and Feingold reported that excess risk associated with residential traffic density was limited to the 0–4 age group (RR = 5.6, 95 % CI 1.9–16.7), while the point estimate for RR for children aged 5–14 was below unity (RR = 0.4, 95 % CI 0.1–2.8) [35]; our results largely mirror these findings. Feychting et al. noted a higher overall childhood cancer relative risk associated with motor vehicle exhaust exposure (estimated by modeling nitrogen dioxide emissions) in the age group 0–5 compared with ages 5–9 and 10–14 [36]. On the other hand, Raaschou-Nielsen et al. [37] did not observe major age-related differences in childhood leukemia risk associated with benzene exposure. In addition, Reynolds et al. [38] did not identify an association between leukemia risk and road or traffic density in a study carried out on children younger than 5 years in California. In a further subgroup analysis for major childhood leukemia subtypes, i.e., acute lymphoblastic leukemia and acute myeloid leukemia, the relation with benzene exposure appeared considerably stronger for the latter category and in the youngest age group, although the estimates were imprecise owing to the small numbers involved. Such observation is of interest due to the already established association in adults with the same leukemia subtype, acute myeloid leukemia [4, 5, 13], but needs to be confirmed in much larger case series.

Previous epidemiologic studies generally based estimated individual exposure to pollutants from traffic on distance from main roads or data from air monitoring stations located in the same (generally broad) areas [4, 31]. These approaches are subject to substantial exposure misclassification, since exposure depends not only on distance but on the numbers and types of vehicles circulating on all nearby major and minor roads and meteorological conditions, data which were generally unavailable in previous studies. While our detailed exposure assessment is a strength, it must be noted that in our study, assessment of PM_10_ exposure was biased towards much lower values than actually measured in the study area since, unlike benzene, motorized traffic is not the major source of PM_10_ even in urban areas. In our investigation, estimated concentrations of PM_10_ at the 7 monitoring stations recording PM_10_ in the study area were 24.4 % of the measured values, a value very similar to that estimated as contribution of traffic to measured environmental levels of PM_10_ or its major component PM_2.5_ in recent studies in Milan (27.1 and 17–24 %, respectively) [39, 40].

Recent studies with a different design also suggested a relation between childhood leukemia and low-dose benzene exposure. One such study was an ecologic study in Texas based on census tract-specific benzene estimates [41]; others examined residence near petrol stations and automotive repair garages [42–45] or hazardous waste sites containing benzene [46]. Another study that used frequency of vehicle refueling by parents in the year before or during pregnancy and use of wood burners as indicators of benzene exposure did not find such association [47].

A distinctive feature of the present study was evaluation of PM_10_ as a potential confounder and an independent risk factor for leukemia, an association not analyzed so far to the best of our knowledge. PM_10_ concentrations may be considered a proxy for a number of contaminants hypothetically involved in the etiology of childhood leukemia and more generally of cancer, such as heavy metals and several volatile organic compounds including dioxins, benzene, benz(a)-pyrene, and 1-3 butadiene [18–21, 48, 49]. In our analyses, the independent association between PM_10_ and the disease was considerably weaker than for benzene but still possibly elevated, a relation that may be worth further study [48]. Mean annual levels of PM_10_ in the study area, as measured by the monitoring stations of the two provinces, were in several cases in the order of 40 μg/m^3^, a value which represents the EU standard [30] and is higher than the World Health Organization guideline of 20 μg/m^3^ [50].

The amount of exposure associated with increased childhood leukemia risk in the present study is not easy to determine, particularly since the induction period between benzene exposure and disease onset is unknown. In our study, we observed an excess risk mainly in the highest exposure categories (≥0.5 μg/m^3^ for average benzene concentration and ≥6 μg/m^3^ for maximum hourly benzene concentration). However, since levels of benzene decreased in the study area during the 12-year study period, and more generally in the nation as a whole, the critical amounts of benzene exposure might well have been those at the beginning of the period or immediately before it (estimated on the order of 5 μg/m^3^ or above), depending on the alleged length of the induction period [51, 52]. We note that the RR estimates were somewhat sensitive to the model specification (categorical versus continuous, untransformed versus log-transformed); however, the various modeling approaches were consistent in suggesting excess risk with higher exposure.

In conclusion, our study in this Italian community indicates that exposure to low environmental benzene concentrations released by motorized traffic may be a risk factor for childhood leukemia among children younger than 5 years of age, and particularly for acute myeloid leukemia, although this result could also reflect confounding from unmeasured traffic pollutants or other factors. Our findings offer limited evidence for a role of PM_10_.
